# Pediatric Tuberculosis: Unraveling Immunity, Clinical Complexities, and Resource-Driven Disparities in the Pursuit of Prevention

**DOI:** 10.3390/vaccines14020119

**Published:** 2026-01-27

**Authors:** Daniel Mashiach, Justin Shon, Raquel Mashiach, Gregory Ayzenberg, Osnat Barazani, Andre Aabedi, Vishwanath Venketaraman

**Affiliations:** College of Osteopathic Medicine of the Pacific, Western University of Health Sciences, Pomona, CA 91766, USA; daniel.mashiach@westernu.edu (D.M.); justin.shon@westernu.edu (J.S.); raquelmashiach12@gmail.com (R.M.); gregory.ayzenberg@westernu.edu (G.A.); osnat.barazani@westernu.edu (O.B.); andre.aabedi@westernu.edu (A.A.)

**Keywords:** pediatric tuberculosis, bacille Calmette–Guérin (BCG), multidrug-resistant tuberculosis, tuberculosis prevention, low-resource settings, pediatric immune response, extrapulmonary tuberculosis, latent tuberculosis infection, vaccine equity, child health disparities

## Abstract

Pediatric tuberculosis (TB) remains a critically underrecognized contributor to global childhood morbidity and mortality, with the highest burden concentrated in low-resource settings. Although children comprise a minority of overall TB cases, mortality is disproportionately high, particularly among those under five years of age, driven largely by delayed diagnosis, inadequate linkage to care, and limited access to effective treatment. The continued rise of pediatric multidrug-resistant TB (MDR-TB), especially in regions with low sociodemographic development, further highlights persistent gaps in current control strategies. This review synthesizes key aspects of pediatric TB pathogenesis and host immune responses that predispose young children to rapid disease progression and severe outcomes, including immune immaturity and paucibacillary infection. We summarize pulmonary and extrapulmonary disease manifestations and identify populations at heightened risk, including children with HIV, malnutrition, type 1 diabetes mellitus, and congenital or treatment-related immunosuppression. Ongoing challenges in diagnosis and treatment are discussed, including limitations of existing microbiologic and immunologic tests, specimen collection constraints, regimen toxicity, and barriers to adherence. Prevention remains central to reducing pediatric TB mortality. We highlight the sustained importance of bacille Calmette–Guérin (BCG) vaccination in preventing severe disease and death, the context-dependent variability in vaccine effectiveness, and the structural and socioeconomic determinants of vaccine coverage. We conclude that integrating equitable vaccine delivery, scalable preventive therapy, and child-adapted diagnostic strategies is essential to meaningfully reduce the global pediatric TB burden.

## 1. Introduction

Tuberculosis (TB) has affected humans and animals across the globe for millennia, with the earliest evidence of *Mycobacterium tuberculosis* traced to 9000-year-old human remains from Atlit, Israel, and 17,000-year-old bison remains from North America [1,2]. *M. tuberculosis* is the primary agent responsible for human TB worldwide; however, other members of the *Mycobacterium tuberculosis* complex (MTBC), including *M. africanum*, *M. bovis*, *M. canettii*, *M. caprae*, *M. microti*, and *M. pinnipedii*, contribute to human infection to a far lesser degree and often in geographically or zoonotically restricted contexts [3,4]. TB remains the leading cause of death globally from a single infectious agent, with an estimated 10.7 million incident cases and over 1 million deaths each year [5].

Although children and adolescents constitute a smaller portion of the global TB burden, they experience a disproportionately fatal impact. Individuals ≤15 years account for 11% of TB cases yet 16% of all deaths, resulting in approximately 172,000 pediatric deaths annually [5]. Particularly concerning is the concentration of mortality among the youngest: four out of five pediatric TB deaths occur in children under 5 years of age, and 96% occur in children who never received treatment, underscoring profound gaps in early diagnosis, linkage to care, and therapeutic access in resource-limited settings [6] (Table 1).

**Table 1:** Countries contributing the largest absolute number of pediatric TB cases.

Country	Resource Ranking	Share of Global TB	Total Pediatric TB Cases *
Democratic Republic of the Congo	1 (most resource-limited)	3.9%	46,000
Nigeria	2	4.8%	57,000
Pakistan	3	6.3%	74,000
Bangladesh	4	3.6%	42,000
Indonesia	5	10%	118,000
Philippines	6	6.8%	80,000
India	7	25%	294,000
China	8 (least resource-limited)	6.5%	77,000

* Total pediatric TB cases were calculated by applying the WHO reported pediatric proportion (~11%) to country specific TB caseloads, resulting in the highest absolute pediatric burden in countries with the largest TB incidence [5].

Pediatric disparities are furthered when examining drug-resistant TB. Multidrug-resistant TB (MDR-TB), defined as resistance to at least isoniazid and rifampin, and extensively drug-resistant TB (XDR-TB), which additionally includes resistance to a fluoroquinolone and at least one of bedaquiline or linezolid, pose an escalating threat to pediatric populations. In 2019 alone, roughly 68,000 MDR-TB new cases occurred among children and adolescents less than 20 years old [7]. The overall global burden of MDR-TB has declined in the general population, largely due to widespread bacille Calmette–Guérin (BCG) vaccination, expansion of preventive strategies, and improved diagnostics and therapeutics; however, this trend has not extended to children and adolescents [5,7,8,9]. Over the past three decades, the global incidence of pediatric MDR-TB has steadily increased, with an estimated annual percentage change of 4.2%, and the rise has been especially pronounced in lower sociodemographic index (SDI) regions [7].

The highest MDR-TB incidence rates are concentrated in Southern sub-Saharan Africa, Eastern Europe, and South Asia, while mortality and disability-adjusted life years (DALYs) remain highest among children under 5, who in 2019 experienced mortality rates of 0.62 per 100,000 and DALY rates exceeding 55 per 100,000 [7]. Notably, adolescents aged 15–19 years demonstrate the highest incidence, whereas children less than 5 years bear the greatest mortality, a dual vulnerability reflecting both transmission dynamics and systemic breakdowns in diagnosis and care [7]. The continued rise in pediatric MDR-TB, particularly in lower SDI regions, indicates that existing public health strategies remain insufficient for this age group.

Together, these data demonstrate that pediatric TB remains a critically underrecognized and under-addressed component of the global TB epidemic. Addressing these gaps requires not only improving diagnostic capacity and treatment access but also fundamentally rethinking public health approaches to detection and care for children and adolescents in the world’s most vulnerable regions. This review aims to synthesize current knowledge on pediatric TB, with a particular emphasis in low-resource settings; examine barriers to effective management; and highlight emerging strategies and innovations that may help close the diagnostic and therapeutic gap for this at-risk population.

## 2. Pathogenesis of *M. tuberculosis* in Children

The pathogenesis of TB involves a complex interaction between the host immune system and virulence mechanisms of *M. tuberculosis*, which is the predominant pathogen within the MTBC [3]. Infection begins when aerosolized bacilli are inhaled and deposited within the alveoli, typically from individuals with active pulmonary disease [10]. Alveolar macrophages phagocytose the organisms; however, *M. tuberculosis* subverts intracellular killing by preventing phagosome–lysosome fusion, enabling intracellular survival and early replication [11].

Within a few weeks, antigen-specific T cells activate macrophages and initiate granuloma formation, which is an organized structure intended to contain bacillary proliferation [12]. In young children, however, granulomas are less mature and structurally less effective, allowing organisms to escape containment and disseminate before robust adaptive immunity develops [13,14,15]. Clinically, early pulmonary disease may manifest with nonspecific respiratory symptoms or hypersensitivity reactions such as fever, erythema nodosum, or phlyctenular conjunctivitis [14,16]. As organisms are trafficked to regional lymph nodes, causing transient hilar or mediastinal lymphadenopathy, they may seed the lymphatic and hematogenous circulation, establishing latent foci in distant organs and priming sites for future complications [17,18,19]. The peripheral lymph nodes and central nervous system (CNS) remain the most frequent extrapulmonary sites in children, with lymphadenitis presenting as cool, non-erythematous, painless cervical lymph node enlargement that can progress to fistulization [17].

Young children, particularly those under 5 years, are uniquely vulnerable due to immature cell-mediated immunity, and are more likely to progress from primary infection to disseminated disease rather than establish stable latency [12,13,14]. Hematogenous spread occurs readily, markedly increasing the risk of miliary TB, which is characterized by diffuse involvement of multiple organs, and TB meningitis, the most severe pediatric TB manifestation which carries a 50% mortality rate [13,14,15,20,21,22]. Early symptoms are nonspecific, such as fever, irritability, anorexia, or mild respiratory and gastrointestinal complaints, and can progress to meningismus, seizures, vomiting, cranial nerve palsies, altered mental status, and signs of raised intracranial pressure as inflammation intensifies [20,21,22]. Further complications such as hydrocephalus, cerebrovascular infarction, coma, and tuberculoma formation may also occur [23].

Beyond CNS involvement, children and adolescents frequently suffer long-term pulmonary sequelae, involving complications that are often underrecognized and inadequately managed in low-resource settings [24,25,26,27]. Importantly, because of immature immunity and typically low bacillary burdens, pediatric TB is often paucibacillary, leading to diagnostic challenges across all modalities and contributing to delayed treatment and accelerated disease progression if unrecognized [28].

## 3. Protective Immune Responses Against *M. tuberculosis* in Children

Protective immune responses against TB in children are reliant on the innate and adaptive immune systems. The innate immune system is the first line of defense which utilizes macrophages and dendritic cells to phagocytose TB and presents the antigens to the adaptive immune system. In children, the immune system is not as robust as compared to adults which results in reduced levels of circulating dendritic cells [13]. Pattern recognition receptors, which are part of the innate immune system and include Toll-like receptors, can trigger an inflammatory response and cytokine production to control the infection. Since the immune system is still immature in children, these mechanisms develop and become stronger over time with age. The cytokines produced during an infection like TNF-α and IL-6, are pro-inflammatory cytokines that mediate the cellular response to the TB infection [29]. Natural killer cells play an integral role in linking innate and adaptive immunity, fighting the infection by releasing interferon-γ and granulysin and playing an important role in the clearance of TB before developing acquired immunity [30].

The adaptive immune system uses T cell-mediated responses to activate macrophages to clear the infection. The protective adaptive immunity centers on CD4+ T cells and Th1 pathways. In children, CD4 T cells are not in abundance, which leads to a greater susceptibility to TB. Through the stimulation of CD4 T cells, which produce IFN-γ and TNF-α, macrophages are activated to kill intracellular mycobacteria [31]. CD8 T cells also play a vital role in protection of children against TB. Through the release of cytokines and cytotoxic molecules, CD8 T cells mark infected cells to undergo apoptosis. The role of regulatory T cells (Tregs) in children is very interesting as it acts to suppress the immune system, which allows the infection to grow while preventing excessive inflammation-mediated tissue damage. It has been shown that children with TB have elevated levels of Tregs [32]. Tregs can impair the function of Th1 cells, leading to impaired microbicidal activity by lowering the release of IFN-γ and other pro-inflammatory cytokines, allowing the infection to persist. On the other hand, while the Tregs lower the immune system’s fighting chance against the infection, they prevent the body from having an overpowering pro-inflammatory state which could lead to tissue damage [33].

In healthy adults, coordinated innate and adaptive immune responses often succeed in restraining *M. tuberculosis*. In children, however, these defenses are still maturing, creating a window in which the pathogen can more readily gain a foothold and spread. This developmental immaturity is central to why TB poses such profound and disproportionate danger in early life.

## 4. Pulmonary and Extrapulmonary TB in Children

There are two primary ways to classify TB: pulmonary and extrapulmonary. Pulmonary tuberculosis (PTB) in children involves the lung parenchyma or the tracheobronchial tree and is considered the more common form of TB. PTB manifests in respiratory symptoms like cough, fever, weight loss, and other respiratory symptoms [34]. Chest X-rays in PTB usually show intrathoracic lymphadenopathy where lung opacities may or may not be present. Hilar and mediastinal lymphadenopathy is usually a tell-tale sign of pediatric PTB. Severe lymph node enlargement could result in airway obstruction, especially in younger children due to their small airways, resulting in hyperinflation or collapse of the lungs [35]. Due to younger children having an immature immune system, there is also a higher likelihood of disseminated infection, which is why early treatment management is vital for better prognosis.

**Figure 1:**
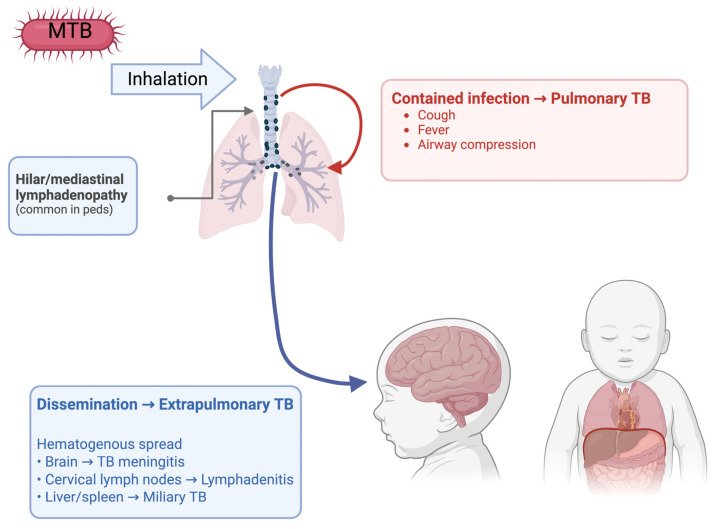
Presentations of pulmonary and extrapulmonary TB in children.

When tuberculosis spreads and infects organs other than the lungs, this is called extrapulmonary tuberculosis (EPTB). EPTB can show up in children in a variety of presentations, such as TB lymphadenitis, TB meningitis, and the worst disseminated form is miliary TB (Figure 1). TB lymphadenitis is the most common form of EPTB in children, which typically shows up as a matted mass of cervical lymph nodes [36]. The lymph nodes are usually painless and constitutional symptoms are usually absent which makes it more difficult to diagnose. Without proper treatment, lymph nodes can grow and form draining sinus tracts; diagnosis is usually achieved through fine needle aspiration cytology [37].

TB may manifest as meningitis with hematogenous spread in children. The typical symptoms in children with TB meningitis are usually nonspecific, such as poor weight gain and a low-grade fever. Diagnosis is usually difficult to catch early on due to nonspecific symptoms and since neck stiffness usually presents later in the progression of the disease [38]. As the infection persists in the brain, the more specific symptoms like altered consciousness, cranial nerve palsies, seizures and increased intracranial pressures are evident. Since the specific symptoms of meningitis are only evident after a persistent infection, the rates of morbidity and mortality are much higher, hence the importance of early diagnosis [39].

Miliary TB in children is the disseminated form of TB which results from a large lymphohematogenous spread of the infection throughout the body. As with other forms of EPTB, the symptoms are usually nonspecific, including cough, fever and weight loss, but can have some more serious symptoms like hepatomegaly and splenomegaly [40]. Diagnosing miliary TB leads to many challenges due to vague clinical presentations, and difficulty obtaining specimens from children. High-resolution CT imaging is used for diagnosis, showing scattered miliary nodules throughout the body [41]. Miliary TB is highly fatal if left untreated, and early diagnosis and treatment with specific anti-TB treatment could be lifesaving, impeding the spread of the infection.

## 5. Pediatric Populations at Heightened Risk for TB

Immature immune systems, environmental exposures, and underlying comorbidities that impair host defenses cause TB to disproportionately affect vulnerable subpopulations. Significant discrepancies between estimated TB incidence in children and reported cases suggest that large numbers of undiagnosed and untreated pediatric TB cases are being missed by health systems [6]. Among these vulnerable subpopulations, young children represent one of the highest-risk groups for severe disease and TB-related mortality.

An estimate of the global TB mortality burden using mathematical modeling across 217 countries revealed that children under 5 years represented 80% of the TB mortality in children. With previous analyses of under-5 mortality critically omitting TB, and over 96% of the TB mortality in children under 5 years not receiving TB treatment, there is ample room for improvement in diagnosis, treatment, and reporting in this population [6,42].

Susceptibility to TB in early childhood is also shaped by nutritional factors that impair host immunity. Undernutrition, defined as a BMI of less than 2 standard deviations below the median, was responsible for an estimated 0.97 million global incident cases of TB compared to 0.93 million cases attributable to diabetes and 0.57 million to HIV infection [5]. Risk is further amplified by environmental and household factors that increase the likelihood of exposure to TB. Children have a higher risk for TB if they live with someone who has TB, recently spent time with someone who has active TB, live in a country with a high burden of TB, or live in a large group setting [5].

In addition to environmental exposure, specific medical conditions that impair immune function substantially increase the risk of TB in children. HIV significantly increases the likelihood, severity, and complexity of TB in children. Diagnostic tools perform less reliably, clinical presentations are more variable, and outcomes are poorer without early ART (antiretroviral therapy) and preventive therapy. Children with HIV are at markedly increased risk for MDR-TB, rapid progression from infection to active and disseminated TB, including severe manifestations such as miliary disease and TB meningitis [43]. Clinical features are often atypical, and extrapulmonary or disseminated presentations occur more frequently than in HIV-uninfected children [43,44,45].

Given this heightened risk and the potential for rapid disease progression, preventive therapy remains a cornerstone of management in children living with HIV. The main healthcare intervention available to reduce the risk of TB infection progressing to active TB disease is preventative treatment. Children with HIV who are exposed to TB should receive treatment for latent TB infection (LTBI) once active disease has been excluded, even when TST (TB skin test) or IGRA (IFN-γ release assay) testing is negative, given the high risk of rapid progression [43]. Short-course rifamycin-based regimens, such as once-weekly isoniazid plus rifapentine for 12 weeks, are preferred when compatible with ART regimens [43,46].

Children living with HIV face markedly elevated risks of tuberculosis, as demonstrated across diverse settings. In a South African cohort of 2219 HIV-infected children ≤5 years, TB incidence reached 28%, with most cases occurring before or shortly after ART initiation. Delayed HIV diagnosis, advanced immunosuppression, and TB were each associated with increased mortality, emphasizing the need for early detection and preventive therapy [44]. Longitudinal data from a national Spanish pediatric HIV cohort similarly showed that, although TB incidence declined with expanded combination ART coverage, TB–HIV coinfection continued to cause substantial morbidity and a 13.8% mortality rate, even in a low-prevalence setting [45]. Complementing these findings, a trial in Cameroon and Kenya found that HIV-infected children under 5 years with TB more often presented with acute malnutrition and experienced higher case fatality (9% vs. 4% in HIV-negative children), underscoring the persistent and heightened vulnerability of HIV-exposed and HIV-infected children to severe TB outcomes [47].

In addition to HIV infection, metabolic disorders that impair immune function have emerged as important risk factors for pediatric TB. Although type 1 diabetes mellitus (T1DM) has traditionally been recognized as a TB risk factor, most research has focused on adults with type 2 diabetes, leaving pediatric T1DM relatively understudied [48]. Emerging evidence now indicates that children and adolescents with T1DM face substantial vulnerability to both TB infection and disease, particularly in high-burden settings where environmental exposure intersects with metabolic instability.

Across sub-Saharan Africa, available studies consistently show a high burden of latent and active TB among children and adolescents with T1DM. In a South African hospital-based cohort, nearly one-third of participants had evidence of TB infection, and the point prevalence of active TB exceeded 3400 per 100,000, far above community rates, with each incremental rise in HbA1c conferring additional risk [49]. A Tanzanian study further demonstrated that LTBI prevalence approached 15% among children and youth with poorly controlled HbA1c, reinforcing the link between hyperglycemia and increased TB susceptibility; demographic factors did not meaningfully modify this risk [50]. Together, these African cohorts point to a consistent pattern: glycemic instability may directly impair host immunity to TB, making optimal diabetes control a potentially modifiable target within pediatric TB prevention strategies.

Evidence from outside Africa aligns with these observations. A large population-based cohort from Taiwan found that children and adolescents with newly diagnosed T1DM had a four-fold higher incidence of TB than matched controls, with risk amplified among those with comorbidities and frequent hospitalizations [51].

Taken collectively, the emerging pediatric literature highlights several themes: children with T1DM exhibit higher-than-expected rates of both LTBI and active TB [49,50,51]; poor glycemic control appears to be a central driver of this vulnerability and is consistent with immunologic studies demonstrating impaired IL-1β responses to TB in T1DM [52]; and, as with all pediatric TB, environmental exposures, particularly household contact with a TB case, remain critical determinants of risk [49].

Children with impaired immune function, whether due to primary immunodeficiencies, rheumatic disease, malignancy, or iatrogenic immunosuppression, are at markedly increased risk for severe and complicated TB. Immunosuppression predisposes patients to disseminated disease, atypical or extrapulmonary presentations, and false-negative immunologic screening tests, making early recognition challenging and delaying treatment initiation [53,54]. Across European cohorts, immunocompromised children consistently demonstrate higher rates of non-respiratory and severe TB than their immunocompetent peers, with significantly elevated frequencies of false-negative TST and IGRA results. These diagnostic limitations highlight the need for symptom-based clinical assessment even when immunologic tests are negative [53].

Host genetics may also define a subset of pediatric patients at heightened risk, particularly those who develop severe or disseminated TB despite limited exposure history or who present at very young ages. Rare, high-impact variants affecting IFN-γ-dependent antimycobacterial immunity have been identified in children with severe TB, and have implicated several genes; however, most genes are yet to be identified [55,56]. Population studies support a more polygenic contribution to TB risk, with common polymorphisms across immune-response genes, including cytokine signaling and antigen-presentation pathways, showing associations that can vary by both ancestry and local epidemiology [55]. Additionally, variation in innate immune effector systems may contribute to early containment differences. Complement pathway gene variation has been associated with pulmonary TB susceptibility, supporting the concept that innate opsonization and inflammatory regulation can influence host vulnerability [57]. Collectively, these findings suggest that, alongside comorbidities and exposure intensity, genetic susceptibility may help explain why a minority of children experience unusually severe disease trajectories.

Children receiving biologic immunosuppression, particularly anti-TNF-α therapy for conditions such as juvenile idiopathic arthritis or Crohn’s disease, exhibit an especially severe TB phenotype. Multicenter analyses show that nearly all TB cases in this population present with disseminated or life-threatening manifestations, including miliary TB and TB meningitis, and that pre-treatment LTBI screening is frequently negative, suggesting that biologics may unmask latent infection or accelerate progression despite standard evaluation [58,59].

Similar patterns are observed among children with rheumatic diseases treated with combinations of corticosteroids, methotrexate, and biologic agents, who exhibit increased LTBI prevalence and a higher likelihood of progression to active disease [60]. Studies of pediatric extrapulmonary TB further identify immune disorders, whether congenital or treatment-related, as major risk factors for complex disease courses, including lymphatic, pleural, and CNS involvement [61].

Primary immunodeficiencies represent another important contributor to severe pediatric TB. Genetic disorders such as Mendelian susceptibility to mycobacterial disease, chronic granulomatous disease, and defects in IFN-γ or IL-12 signaling can impair macrophage function and granuloma formation, predisposing affected children to disseminated or atypical TB presentations [54]. Immunosuppressive medications, particularly corticosteroids and anti-TNF-α agents, can accelerate progression from latent infection to severe disease, sometimes despite routine screening. Primary immunodeficiencies further compound these risks by altering fundamental immune pathways required for TB control.

## 6. Clinical and Laboratory Diagnosis of Pediatric TB

The clinical assessment of TB in children is problematic because patients often present with nonspecific symptoms including poor growth, diminished appetite, and occasional low-grade fever [16]. These findings frequently overlap with other conditions, such as malnutrition, making diagnosis more complex. A persistent cough remains the primary symptom prompting clinical suspicion [62]. Confirmation typically relies on microbiologic and radiologic evidence demonstrating the presence of *M. tuberculosis* [62].

Children typically have low bacillary burden and often cannot expectorate sputum, which limits the reliability of standard microbiologic tests. Gastric aspiration or sputum induction are two alternative and safe procedures to collect respiratory specimens of *M. Tuberculosis* [63]. Culture yield varies with the specimen type, and for this reason induced sputum is considered as a more dependable sample, typically producing higher diagnostic returns due to better specimen quality [63]. Although detection of *M. tuberculosis* nucleic acids demonstrates high specificity by identifying DNA sequences unique to TB, the results are often low in children because of their paucibacillary disease [63]. Molecular tests like Xpert MTB/RIF are useful to detect rifampicin resistance and provide quick results; however, they are less sensitive to detection of *M. tuberculosis* in both children and adults [64]. As a result, many pediatric cases yield negative microbiologic results, making imaging findings and clinical evaluation essential for establishing a diagnosis.

In practice, physicians place the greatest emphasis on pulmonary features, even in cases of extrapulmonary involvement. Radiologic evaluation of pediatric TB presents several challenges, mainly because children show patterns that differ significantly from the classic presentations seen in adults. The most distinctive abnormalities on a chest radiograph (CXR) of pediatric TB are hilar and mediastinal lymphadenopathy, which are significantly less common in adults [65,66]. These findings are subtle because the CXR appearance can resemble pneumonia, which is a more common infection in children [67]. This similarity may lead to misdiagnosis of TB as a simple infection, delaying proper treatment. Limitations of relying solely on CXR include reader disagreement due to the low specificity of radiographic findings. Miliary TB is often better detected by computed tomography than CXR because of the scattered distribution of tiny, innumerable 1–2 mm nodules across both lungs [68]. Ultimately, the variability and elusiveness of these imaging patterns underscore the need for a comprehensive diagnostic approach beyond radiology.

**Figure 2:**
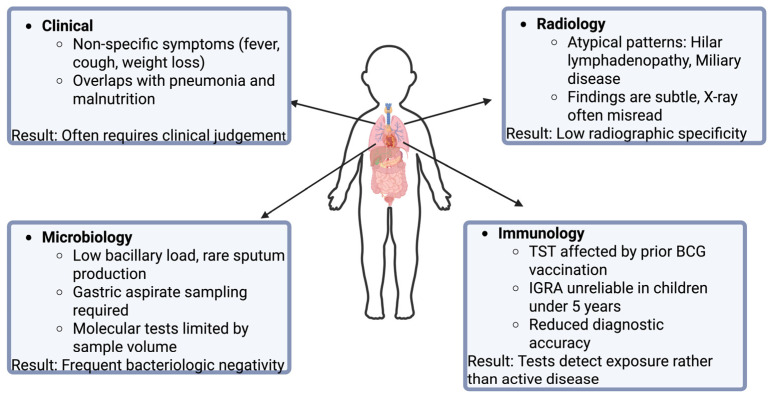
Challenges in the diagnosis of pediatric TB.

The diagnostic accuracy of immunologic tests for tuberculosis is reduced in pediatric populations, making their interpretation more complex than in adults. As an initial concern, TST can be difficult to administer accurately in a squirming child compared with a cooperative adult. In children, viral illnesses may temporarily suppress the immune response, producing false-negative TST results, whereas prior BCG vaccination may lead to false-positive reactions in approximately half of those tested [69]. This effect generally wanes after three years but testing sooner than three years post-vaccination is more likely to yield false positives, and repeated TSTs can boost the immune response, further increasing this risk [69,70,71]. Beyond these factors, both TSTs and IGRAs have reduced sensitivity in children under five years old, increasing the chance of false-negative results. Thus, a negative test cannot reliably exclude infection, while a positive IGRA in this age group usually indicates true infection [69,72]. Although fewer studies exist on IGRAs in children than in adults, available evidence suggests these tests perform reliably in children aged four and older [72,73]. Importantly, neither TSTs nor IGRAs can distinguish between LTBI and active disease [69]. These limitations highlight the need to combine test results with clinical assessment and exposure history in pediatric TB (Figure 2).

## 7. Treatment of Pediatric TB and Difficulties

The global success rate of drug-susceptible TB treatment has increased over the past couple years from 86% in 2020 to 88% in 2023 [5]. Among the pediatric population specifically, the treatment success rate of drug-susceptible TB has slightly increased from 90% in 2022 to 92% in 2023 [5]. Despite this, international efforts are being made to reduce the impact of TB on the pediatric population by increasing access and adherence to TB medications [5,74].

For non-severe, drug-susceptible TB, a combination of treatments are used as follows: isoniazid (H) 7–15 mg/kg, rifampin (R) 10–20 mg/kg, pyrazinamide (Z) 30–40 mg/kg, and ethambutol (E) 15–25 mg/kg [75,76]. A 6-month regimen of HRZE, consisting of 2 months of HRZE and 4 months of HR, was effective and recommended by the U.S. Centers for Disease Control and Prevention and the Infectious Diseases Society of America [76,77]. However, a more recent SHINE trial revealed that the use of a 4-month regimen of HRZE, consisting of a 2-month regimen of HRZE and 2 months of HR, was equivalent to the 6-month regimen [35,76]. Importantly, this shortened regimen is not recommended for pediatric patients with severe, drug-susceptible pulmonary TB, and a 6-month regimen of HRZE is still recommended as the initial treatment approach [35]. Other forms of TB also exist, necessitating a different combination of medications. Extrapulmonary TB, such as tuberculous meningitis, requires a different combination of drugs: 2 months of HRZ with an aminoglycoside or ethionamide followed by 7–10 months of HR [35]. Not only do variations in TB presentation alter treatment regimens, but drug-resistant forms of TB require changes in treatment approach.

MDR-TB is characterized by the bacteria’s resistance to anti-TB medications. In 2024, the WHO estimated the number of MDR-TB and rifampin-resistant TB (RR-TB) to be 390,000 in the total population [5]. Drug-resistant strains have a significant economic impact. In fact, fluoroquinolone-resistant RR-TB strains cost three times more than fluoroquinolone-susceptible strains [78]. MDR-TB also affects timelines and quality of treatment. Pediatric patients treated with MDR-TB required significantly longer treatment durations and had lower cure rates (67.4% vs. 92.5%) compared to drug-susceptible groups [79]. However, non-first line medications, such as the combination of bedaquillin, pretomanid, and linezolid, have given an effective method in combating RR-TB strains among adolescents [77,80].

Despite substantial advances in tuberculosis therapeutics, significant challenges persist. Anti-TB regimens are frequently associated with adverse effects ranging from mild reactions, such as fever and rash, to severe complications including hepatotoxicity and renal impairment [81,82]. These toxicities substantially undermine treatment adherence, with medication-related adverse events consistently identified as a key predictor of noncompliance [83]. Shorter treatment durations have been shown to improve adherence and treatment completion rates [84], underscoring the importance of ongoing efforts to optimize and shorten therapeutic regimens without compromising efficacy.

## 8. Preventing *M. tuberculosis* Infection

Preventative therapy is one of the most effective tools available to reduce the burden of pediatric tuberculosis, especially in young children with recent TB exposure and those with HIV. Evidence from multiple cohort studies and meta-analyses consistently demonstrates that isoniazid or rifamycin-based regimens substantially lower the risk of progression to disease, with the greatest benefit observed in children under five years of age and in those with confirmed TB [85,86]. Despite its proven efficacy, however, implementation of preventive therapy remains uneven, with major gaps in contact investigation, therapy initiation, and treatment completion across high-burden regions [87,88].

Early diagnosis, though critical, remains challenging. For example, both the TST and IGRA may have reduced sensitivity in the setting of immunosuppression, particularly in children with low CD4 counts. While either test can be used for screening, neither reliably excludes TB, making careful assessment of exposure history and clinical findings essential [43].

Community-based data show that children who received isoniazid preventive therapy were 82% less likely to develop TB than those who did not [85]. Yet, even within a well-supported program, only about three-quarters of eligible children initiated treatment. Preventive therapy can achieve high completion rates of up to 94% with when families receive short-course rifampicin–isoniazid regimens, transportation support, follow-up reminders, and streamlined care pathways [88].

Meta-analytic evidence further clarifies which groups benefit most from preventive treatment. Among children with confirmed TB infection or recent exposure, the number of children who need to receive treatment to prevent one case of disease can be as low as 10–40 in high-burden settings [86]. However, another large individual-participant meta-analysis found that the majority of childhood TB occurs within weeks of exposure, often before preventive therapy can be initiated [89].

Although household exposure to TB source cases is strongly correlated with TB acquisition [49], low rates of household contact investigation limit the number of children identified early enough to benefit from preventive therapy [87,89]. Even when children are reached, caregivers often express reluctance to administer medication to an asymptomatic child, particularly when therapy is lengthy, unpleasant, or when health system messaging is unclear. Treatment duration, medication palatability, and follow-up requirements create additional obstacles to adherence [87]. Children in multiple settings have reported difficulty swallowing pills or repulsion to medication taste, while caregivers express concern about potential side effects despite reassurance from clinicians. These issues are especially relevant for preventive therapy, where medications are given to children who are not ill and therefore may have limited motivation to continue treatment [90,91].

Short-course regimens offer promising solutions, yet access to these child-friendly formulations remains inconsistent, particularly in resource-limited settings [85,88]. Finally, the absence of rapid point-of-care tools to confirm infection in young children contributes to treatment delays, as health systems often rely on slow or incomplete screening pathways [87]. Faster, less resource-intensive diagnostic testing, such as stool samples and CXR with artificial intelligence-based interpretation, are being assessed with promising results for low-resource areas [92].

Effective patient and caregiver education is central to successful pediatric TB prevention and treatment, yet research demonstrates that families often face substantial barriers to understanding, trusting, and engaging with TB care. Across diverse settings, challenges such as limited TB knowledge, stigma, cultural beliefs, language barriers, and mistrust of health systems impede preventive therapy and adherence to treatment recommendations [93,94]. Structural factors, including financial constraints, transportation difficulties, and fragmented health services, compound treatment and follow-up problems.

Qualitative work in both high and low burden settings shows that caregivers commonly have limited understanding of TB infection, its risks, and the purpose of preventive treatment. Misinterpretation of latent TB infection as “not a real disease,” fear of medication side effects, and reluctance to treat asymptomatic children contribute to poor engagement and low adherence to therapy [90,93]. Many caregivers struggle to recognize the urgency of treatment in children who appear healthy, leading to delays or refusal of recommended preventive regimens. In addition, logistical burdens such as time away from work, transportation challenges, and competing childcare responsibilities further limit caregivers’ ability to attend educational sessions or adhere to follow-up appointments [93].

Cultural context plays a major role in shaping families’ perceptions of TB and influencing their decisions regarding care. Research from southern Mozambique reveals that caregivers often attribute TB to traditional or spiritual causes, rely on non-biomedical treatments, or view TB as a consequence of social transgressions. These beliefs contribute to delayed care-seeking, skepticism toward diagnostic procedures, and inconsistent treatment adherence [95]. Similarly, studies among immigrant and indigenous communities in Canada show that language barriers, lack of culturally appropriate education, and negative attitudes toward TB significantly disrupt prevention and treatment efforts [96]. Systematic reviews confirm that stigma, cultural misconceptions, and historical mistrust of health systems remain pervasive obstacles to effective education, especially among migrant and hard-to-reach populations [94]. Communication challenges between families and healthcare providers further complicate education and adherence. Pediatric TB prevention requires multidirectional communication, not only between clinicians and caregivers but also involving the child, yet families frequently report poor clarity, inconsistent messaging, and difficulty navigating complex medical instructions [87,93]. Age-appropriate counseling, visual aids, simplified explanations, and bilingual support are often lacking, making it difficult for caregivers and older children to understand the rationale for preventive therapy or the importance of completing treatment. Studies emphasize that health education must be tailored to the child’s developmental stage and the family’s cultural and linguistic needs to be effective.

Addressing these difficulties requires culturally sensitive, family-centered education that incorporates community engagement, multilingual communication, trust-building strategies, and developmentally appropriate tools for children. Without such efforts, even the most effective TB prevention and treatment strategies will continue to fall short in real-world pediatric populations.

## 9. BCG Vaccine

The BCG vaccine was first administered to a neonate in 1921 and remains the most widely used vaccine worldwide [97]. To date, more than four billion doses have been administered, and as of 2024, approximately 88% of children globally receive BCG during infancy [5,98]. Beyond its widespread uptake, BCG has demonstrated substantial clinical benefit, conferring an estimated 71% reduction in tuberculosis-related mortality among vaccinated children [99].

BCG is a live attenuated strain of *Mycobacterium bovis* that exerts its protective effects through both innate and adaptive immune mechanisms. Following administration, mycobacterial components are recognized by pattern recognition receptors, including Toll-like receptors 2 and 4 on macrophages, initiating a robust inflammatory response [100]. This signaling cascade promotes the production of pro-inflammatory cytokines such as TNF–α and IL-1β, facilitating dendritic cell maturation and antigen presentation [101,102]. In neonates, BCG vaccination further drives T-helper 1 polarization, which is characterized by increased IFN-γ production and acts as a critical mediator of antimycobacterial immunity [102,103]. Emerging evidence also highlights epigenetic reprogramming as a key component of BCG-induced immunity, with increased histone trimethylation enhancing long-term pro-inflammatory cytokine responses, which is a phenomenon consistent with trained innate immunity [104].

Despite its well-established benefits, BCG vaccination is not without risk. Adverse events include regional lymphadenitis, osteomyelitis, and localized injection-site reactions, and rare cases of disseminated BCG disease have been reported in immunocompromised children [105,106]. Nonetheless, population-level data underscore the profound consequences of withholding vaccination. A temporary one-year suspension of BCG administration in Bosnia and Herzegovina in 2003 was associated with a 175-fold increase in neonatal tuberculosis meningitis, illustrating the critical protective role of continued vaccination programs [107]. These findings highlight the necessity of carefully balancing individual-level risks against substantial public health benefits.

BCG vaccine effectiveness is also known to vary by geographic and immunologic context. Randomized controlled trials comparing adolescents in Malawi and the United Kingdom demonstrated diminished post-vaccination immune responses among individuals with higher baseline mycobacterial exposure [108]. Consistent with these observations, a meta-analysis revealed greater vaccine-derived protection in populations residing farther from the equator, where exposure to environmental nontuberculous mycobacteria is less prevalent [109]. These data support the hypothesis that prior mycobacterial sensitization may attenuate BCG-induced immune responses.

Nevertheless, optimizing BCG coverage remains a critical priority, particularly in regions with persistently low vaccination rates. Observational data from Guinea-Bissau indicate that children born in healthcare facilities are significantly more likely to receive BCG vaccination than those born at home, while markers of socioeconomic disadvantage, including lack of mobile phone access and inadequate sanitation, are strongly associated with under-vaccination [110]. Importantly, following the implementation of monthly village outreach visits, 91% of mothers of unvaccinated children were aware of their child’s scheduled vaccination, suggesting that missed immunization opportunities are driven less by knowledge gaps and more by structural barriers. Financial constraints (61%) and geographic inaccessibility of vaccination sites (39%) were identified as the leading causes of non-vaccination [110]. Collectively, these findings underscore that socioeconomic and infrastructural barriers, rather than vaccine hesitancy, remain the dominant determinants of BCG coverage in low-resource settings.

## 10. Conclusions

Although children and adolescents account for a smaller fraction of the global TB caseload, they shoulder a disproportionate burden of mortality, most acutely in early childhood, where deaths overwhelmingly occur among children who never receive treatment, reflecting persistent failures in timely detection, linkage to care, and access to effective therapy in resource-limited settings [6]. The sustained rise of pediatric MDR-TB, particularly in lower SDI regions, further underscores that current approaches have not adequately protected this population [7]. These inequities intensify in the presence of comorbidities such as HIV, T1DM, and other forms of immunosuppression, in which diagnostic sensitivity is reduced, clinical phenotypes are more severe, and health-system barriers compound delays in treatment initiation [43,44,45,46,47,48,49,50,51,52,53,54,55,56,57,58,59]. Together, these patterns point to a persistent and preventable gap: pediatric TB remains concentrated where diagnostic infrastructure is weakest and where children are least likely to be reached by preventive and therapeutic services. These failures are not solely biomedical, they are political, shaped by governance, financing priorities, and the unequal allocation of diagnostics, medicines, and health workforce capacity. Accordingly, the most immediate gains will come from humanitarian, equity-centered delivery: strengthening frontline systems, protecting access for marginalized regions, and ensuring that proven tools are accessible to children in a timely manner.

Vaccination remains the most powerful population-level tool for reducing severe pediatric TB outcomes, and the continued centrality of BCG in child survival should be explicit in TB elimination strategies. BCG’s long-standing protective effect against severe disease and TB-related mortality reinforces the public health imperative of maintaining high infant coverage, particularly in settings where young children face intense exposure and limited access to advanced diagnostics [97,98,99]. Importantly, policy decisions regarding BCG cannot be evaluated solely through an individual risk lens: although adverse events occur and disseminated BCG disease is a rare but serious concern in immunocompromised children [105,106], population-level consequences of disrupted immunization can be catastrophic, as illustrated by the marked increase in neonatal TB meningitis following temporary program suspension [107]. At the same time, heterogeneity in BCG effectiveness, potentially shaped by environmental nontuberculous mycobacterial exposure and baseline mycobacterial sensitization, highlights that vaccine impact is context dependent and that future vaccine strategies must be evaluated across diverse epidemiologic settings rather than extrapolated from single regions [108,109]. In this way, BCG is both a lifesaving intervention and a reminder that biological efficacy and real-world effectiveness are inseparable from context, delivery systems, and equity.

**Figure 3:**
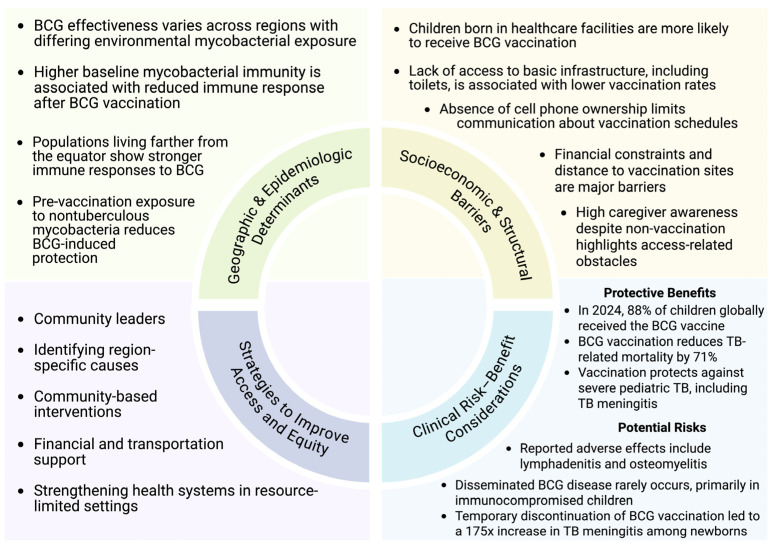
Determinants and strategies influencing pediatric vaccination coverage.

The path forward therefore requires integrating biomedical advances with implementation strategies that address structural determinants of access. Historically, global TB control has emphasized vertical interventions, more sensitive diagnostics, shorter-course preventive therapy, and newer treatment regimens [111]. These remain essential, particularly for pediatric TB where paucibacillary disease and specimen constraints undermine conventional testing, and where regimen toxicity and treatment duration continue to erode adherence. However, in the highest-burden low-SDI settings, vertical strategies alone are insufficient when the primary drivers of missed diagnosis and incomplete care include distance to services, cost, fragile supply chains, stigma, and limited trust in health systems [111,112,113,114]. Pediatric TB control must be built as a family-centered, community-embedded program that pairs diagnostic and therapeutic innovation with locally informed delivery models (Figure 3).

This review highlights several practical priorities with immediate relevance to low-resource implementation. First, preventive therapy must be scaled as a default pediatric protection strategy, not as an aspirational add-on: short-course rifamycin-based regimens can achieve very high completion when paired with transportation support, follow-up reminders, child-friendly formulations, and streamlined pathways that minimize repeated clinic visits [88]. Second, diagnostic strategies should be explicitly adapted to the realities of childhood TB and constrained laboratory capacity, prioritizing feasible specimen collection and decentralization. Approaches such as stool-based testing and simplified imaging workflows, including computer-assisted interpretation where feasible, offer promising routes to reduce reliance on sputum and increase diagnostic reach in young children [92]. Third, pediatric TB risk stratification should be more proactive: in high-burden settings, age-anchored screening touchpoints (e.g., infancy, early childhood, and adolescence) and focused screening in high-risk groups (HIV, diabetes, immunosuppressed children) could reduce the interval between exposure and intervention, particularly given evidence that many pediatric cases progress rapidly after exposure [89].

Finally, equity in vaccine delivery is not merely a coverage metric; it is a system performance indicator. The same barriers that limit BCG uptake, home birth, poverty-linked access constraints, and distance to services, also restrict timely diagnosis and completion of preventive therapy [110]. The Guinea-Bissau data are instructive: high caregiver awareness after outreach suggests that under-vaccination frequently reflects structural barriers rather than hesitancy, with costs and geographic inaccessibility dominating reasons for missed immunization [110]. Translating this into policy implies that strengthening BCG programs should be coupled with investments in outreach infrastructure (e.g., community health worker networks, mobile clinics, predictable supply chains, and integrated maternal–child health services). Community leaders and trusted messengers are not adjuncts to TB programs; they are the essential interface through which vaccination, screening, preventive therapy, and stigma reduction become feasible and sustainable. Immunization strategies must be evaluated and deployed with an explicit commitment to implementation equity, ensuring that scientific progress translates into mortality reduction where the burden is greatest.

In summary, reducing pediatric TB deaths in the coming decade will require aligning vaccine strategy, preventive therapy, and child-adapted diagnostics with delivery systems built for low-resource contexts. Sustained BCG coverage, targeted screening of high-risk children, and simplified, family-centered preventive therapy are immediate opportunities to avert severe disease and death, while next-generation vaccines and regimen innovations must be pursued with equity-first deployment models. A pediatric TB agenda that is age-specific, regionally tailored, and structurally informed is not only scientifically warranted, it is also the most direct route to closing the persistent survival gap for children in high-burden settings.

## Data Availability

The original contributions presented in this study are included in the article. Further inquiries can be directed to the corresponding author.

## Abbreviations

The following abbreviations are used in this manuscript:

TB	Tuberculosis
MTBC	*Mycobacterium tuberculosis* complex
MDR-TB	Multidrug-resistant TB
XDR-TB	Extensively drug-resistant TB
BCG	Bacillus Calmette–Guerin
SDI	Sociodemographic index
DALY	Disability-adjusted life year
Treg	Regulatory T cell
CNS	Central nervous system
PTB	Pulmonary tuberculosis
EPTB	Extrapulmonary tuberculosis
LTBI	Latent TB infection
TST	TB skin test
IGRA	IFN-γ release assay
ART	Antiretroviral therapy
T1DM	Type 1 diabetes mellitus
CXR	Chest radiograph
H	Isoniazid
R	Rifampin
Z	Pyrazinamide
E	Ethambutol
RR-TB	Rifampin-resistant TB
